# Chimpanzee population structure in Cameroon and Nigeria is associated with habitat variation that may be lost under climate change

**DOI:** 10.1186/s12862-014-0275-z

**Published:** 2015-01-21

**Authors:** Paul R Sesink Clee, Ekwoge E Abwe, Ruffin D Ambahe, Nicola M Anthony, Roger Fotso, Sabrina Locatelli, Fiona Maisels, Matthew W Mitchell, Bethan J Morgan, Amy A Pokempner, Mary Katherine Gonder

**Affiliations:** Department of Biology, Drexel University, Philadelphia, PA 19104 USA; Department of Biological Sciences, University at Albany – State University of New York, Albany, NY 12222 USA; Institute for Conservation Research, Zoological Society of San Diego, Escondido, CA 92027 USA; Ebo Forest Research Project, BP 3055, Messa, Yaoundé, Cameroon; Wildlife Conservation Society – Cameroon, Yaoundé, Cameroon; Department of Biological Sciences, University of New Orleans, New Orleans, LA 70148 USA; Institut de Recherche pour le Développement (IRD) and Université Montpellier 1 (UM1), Montpellier, 34394 France; School of Natural Sciences, University of Stirling, Stirling, FK9 4LA UK; Wildlife Conservation Society, Bronx, New York 10460 USA

## Abstract

**Background:**

The Nigeria-Cameroon chimpanzee (*Pan troglodytes ellioti)* is found in the Gulf of Guinea biodiversity hotspot located in western equatorial Africa. This subspecies is threatened by habitat fragmentation due to logging and agricultural development, hunting for the bushmeat trade, and possibly climate change. Although *P. t. ellioti* appears to be geographically separated from the neighboring central chimpanzee (*P. t. troglodytes*) by the Sanaga River, recent population genetics studies of chimpanzees from across this region suggest that additional factors may also be important in their separation. The main aims of this study were: 1) to model the distribution of suitable habitat for *P. t. ellioti* across Cameroon and Nigeria, and *P. t. troglodytes* in southern Cameroon, 2) to determine which environmental factors best predict their optimal habitats, and 3) to compare modeled niches and test for their levels of divergence from one another. A final aim of this study was to examine the ways that climate change might impact suitable chimpanzee habitat across the region under various scenarios.

**Results:**

Ecological niche models (ENMs) were created using the software package Maxent for the three populations of chimpanzees that have been inferred to exist in Cameroon and eastern Nigeria: (*i*) *P. t. troglodytes* in southern Cameroon, (*ii*) *P. t. ellioti* in northwestern Cameroon, and (*iii*) *P. t. ellioti* in central Cameroon. ENMs for each population were compared using the niche comparison test in ENMtools, which revealed complete niche divergence with very little geographic overlap of suitable habitat between populations.

**Conclusions:**

These findings suggest that a positive relationship may exist between environmental variation and the partitioning of genetic variation found in chimpanzees across this region. ENMs for each population were also projected under three different climate change scenarios for years 2020, 2050, and 2080. Suitable habitat of *P. t. ellioti* in northwest Cameroon / eastern Nigeria is expected to remain largely unchanged through 2080 in all considered scenarios. In contrast, *P. t. ellioti* in central Cameroon, which represents half of the population of this subspecies, is expected to experience drastic reductions in its ecotone habitat over the coming century.

**Electronic supplementary material:**

The online version of this article (doi:10.1186/s12862-014-0275-z) contains supplementary material, which is available to authorized users.

## Background

**Figure 1:**
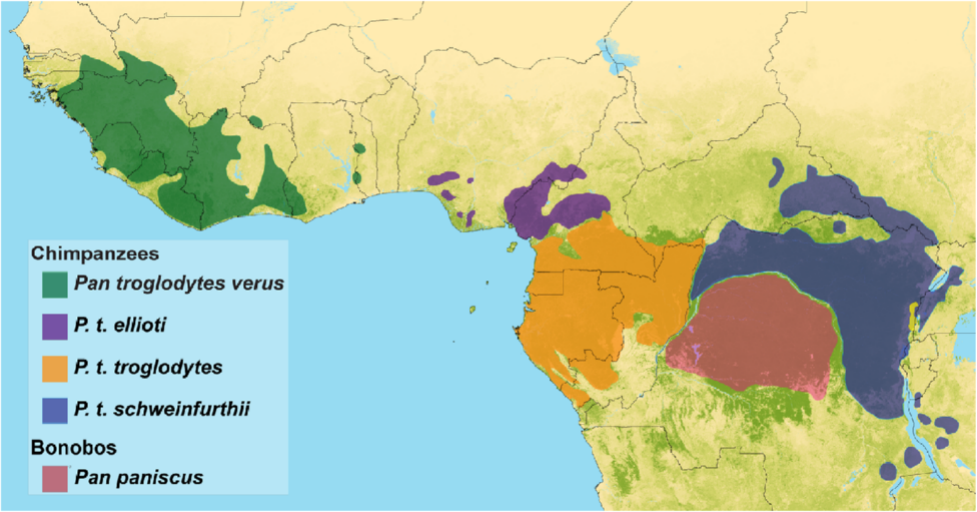
**Chimpanzee subspecies ranges.** Distribution of the genus *Pan*, including bonobos and the four subspecies of chimpanzee.

Chimpanzees and bonobos belong to the genus *Pan*. Bonobos (*Pan pansicus*) occupy the dense wet forests south of the Congo River, whereas chimpanzees (*P. troglodytes*) occupy a much broader range of forested habitats located north of the Congo River and across equatorial Africa [1-4] (Figure 1). Chimpanzees are widely considered to be divided into four subspecies [1-5]: *P. t. verus* occurs in the Upper Guinea region of western Africa; *P. t. ellioti* has a patchy distribution spanning from western Nigeria to central Cameroon; *P. t. troglodytes* occupies the Congo Basin with a range that spans from southern Cameroon and eastward to the Ubangi River; *P. t. schweinfurthii* occupies forests east of the Ubangi River to the Rift Valley [6].

Recently Junker *et al.* [7] created ecological niche models (ENMs) for all African great apes. This study was comprehensive, and included two composite maps of taxon-specific ENMs for each ape subspecies at a continental scale. They compared ENMs from the 1990s and early 2000s in order to determine which ape taxa had experienced the most significant loss of suitable habitat in the recent past. For chimpanzees, they reported that *P. t. verus* and *P. t. troglodytes* had experienced the most significant decline in suitable habitat, but that *P. t. ellioti* and *P. t. schweinfurthii* have not experienced any significant changes in suitable habitat [7]. These conclusions were attributed to previous widespread habitat loss in *P. t. ellioti* and *P. t. schweinfurthii*. However, this study was carried out at a coarse resolution on a continental scale and did not account for two important factors, namely 1) the detailed population genetic structure of chimpanzees across Africa, particularly Cameroon, and 2) the potential role of future climate change on the distribution of suitable habitat for the two chimpanzee subspecies that occupy this region. Accounting for both of these factors in ENMs is important for at least two reasons. First, it is unknown what role niche divergence plays in the genetic divergence of chimpanzees, or even if the various chimpanzee subspecies occupy significantly different types of habitats. Data regarding the relationship between the distribution of genetic diversity and environmental variation remain sparse for the study region, but a growing body of evidence suggests that a strong relationship exists between the partitioning of adaptive genetic variation and environmental variation in Cameroon for the few taxa studied to date [8-10]. Second, this region of Africa is expected to experience dramatic changes in forest cover and composition in response to climate change, and these changes are expected to accelerate over the next century [11-15].

### Study area and taxa

**Figure 2:**
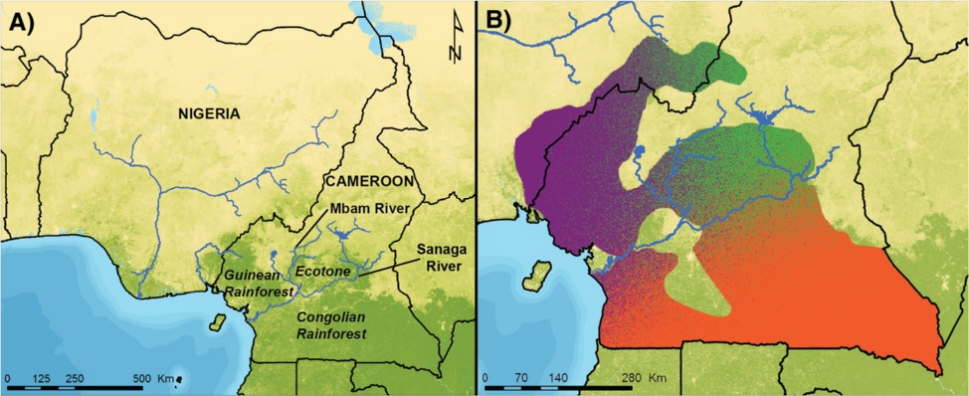
**Habitat types and chimpanzee population history in Cameroon and Nigeria. A**. Different habitat types and major rivers across Cameroon and Nigeria **B**. Population history of chimpanzees in Cameroon and adjacent parts of Nigeria inferred from the analysis of 21 autosomal microsatellite loci in 187 unrelated chimpanzees [27].

The Gulf of Guinea region of Africa is widely recognized as a biodiversity hotspot of global significance due to the region’s high number of endemic taxa [16,17]. The reasons why this region has so many endemic taxa remain unclear, but this pattern of high endemism has been attributed to the effects of geographic barriers, such as the Niger River, Sanaga River, and the Cameroon Highlands, as well as to the history of the forests in this area during the Pleistocene [1,16,18,19]. This area also includes a conspicuous transition between three major biomes. Specifically, the Gulf of Guinea rainforest and the Congolian rainforest biomes converge with each other and with open savanna [20,21]. These three habitats meet in central Cameroon, forming an ecotone comprised of a forest-woodland-savanna mosaic (Figure 2A). Ecotones across the world are increasingly recognized as being important in driving variation in a number of taxa [22,23], and this ecotone in Cameroon has been shown to be important in driving diversification in insects, reptiles, and birds [8-10].

With respect to chimpanzees, Cameroon is unique because it is home to two of the four subspecies: *P. t. ellioti* (the Nigeria-Cameroon chimpanzee) and *P. t. troglodytes* (the Central chimpanzee). The ranges of these two subspecies meet along the Sanaga River in central Cameroon, which has been proposed to separate them [3,24-26]. The Sanaga River is also notable because it has been proposed to influence the distributions of several other species that occupy different niches including, *Mandrillus leucophaeus*/*M. sphinx*, *Cercopithecus erythrotis*/*C. cephus*, *C. nictitans martini*/*C. n. nictitans*, and *C. pogonias pogonias*/*C. p. grayi* [1,16,18,19,24]. A clearer understanding of the role that environmental variation has played in delimiting the distribution of chimpanzee subspecies across this region may help to clarify why this region plays an important role in shaping the distribution of other forest-dwelling primates.

Figure 2B shows the population structure of chimpanzees from this region inferred by fine scale population sampling and genetic analysis of wild chimpanzees [27] suggesting that chimpanzees across this region are divided into as many as three distinct populations. A primary division of chimpanzee populations occurs at the Sanaga River, which separates *P. t. troglodytes* in southern Cameroon from *P. t. ellioti* in central and western Cameroon north of the Sanaga. In addition, *P. t. ellioti* may be further subdivided into two additional populations: one in the rainforests of western Cameroon, which is separated from the second population located in the ecotone habitat [9] east of the Mbam River (Figure 2A). For convenience, these three chimpanzee demes are called the *P. t. ellioti* (Rainforest) population (shown in purple in Figure 2B), the *P. t. ellioti* (Ecotone) population (shown in green in Figure 2B) and the *P. t. troglodytes* population (shown in orange in Figure 2B) throughout this document.

While this region of Africa appears to be an engine of diversification in chimpanzees, the proximate mechanisms that make this region so important for this species remain unclear [8-10]. The Sanaga River lies in the area where the Gulf of Guinea rainforest meets the Congo Basin rainforest, and its headwaters are in an ecotone in central Cameroon. This complexity makes it difficult to attribute the separation of these taxa solely to their separation along the banks of the Sanaga, particularly since habitat variation across this region appears to influence the distribution of other taxa that occupy vastly different niches [1,16,18,19,24]. These observations suggest that ENMs predicated upon the population genetic structure of chimpanzees across the region on a fine geographic scale may help resolve the role that habitat variation plays in delimiting the distributions of chimpanzees in the Gulf of Guinea and Congo Basin forests. In addition, ENMs made at a fine geographic scale may be more useful than continental-scale models (i.e., Junker et al. [7]) for more fully understanding future threats to these populations. Specifically, this study was designed to address two key questions: (1) Do the genetically defined populations of chimpanzees across this region occupy significantly different habitats and if so, which environmental factors appear to be the most important in describing suitable habitat for each population? (2) If a relationship exists between environmental variation and the partitioning of genetic variation, will ongoing and future climate change contribute to altering the remaining distribution of their suitable habitat(s)?

## Results and discussion

### Maxent modeling under present conditions

**Figure 3:**
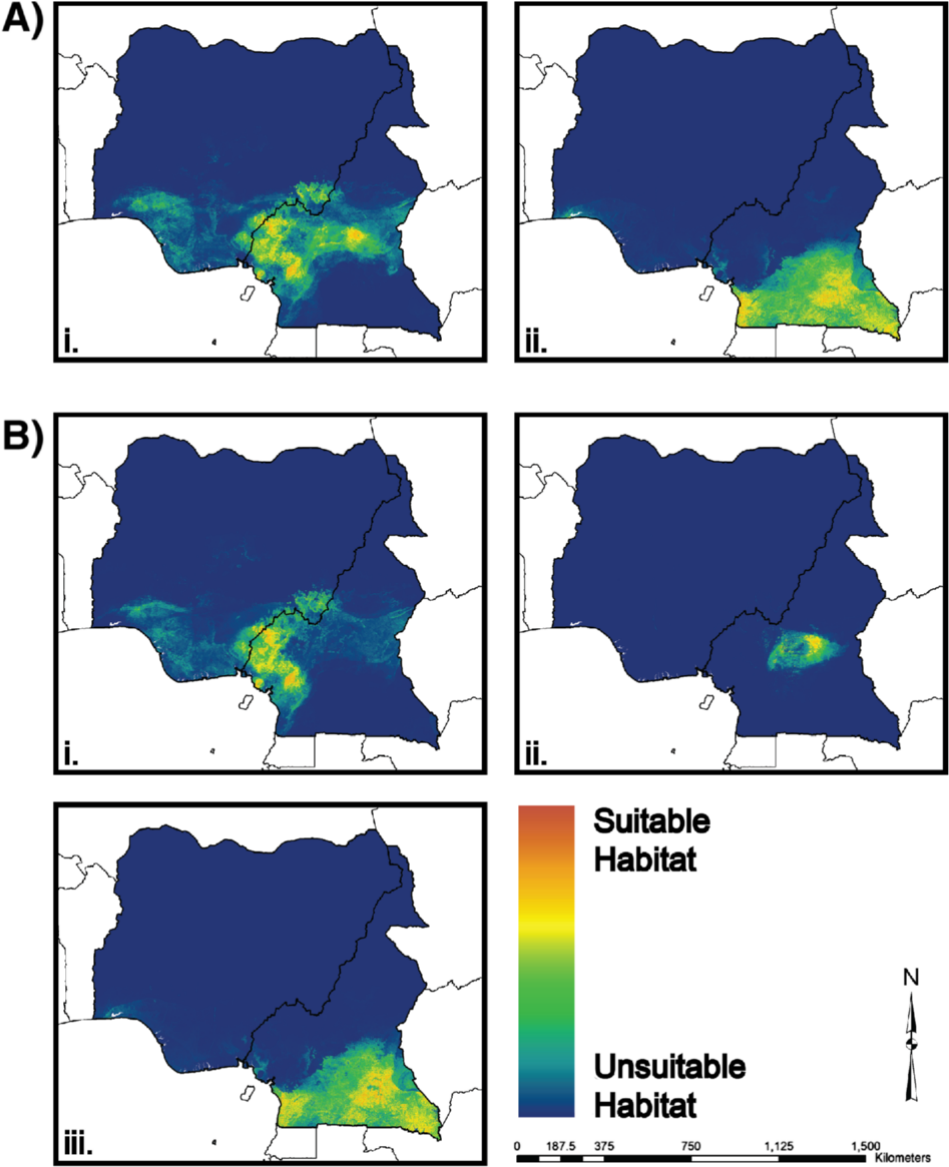
**Ecological niche models for chimpanzee populations in Cameroon and Nigeria. A**. Two-population model: (*i*) *P. t. ellioti*, (*ii*) *P. t. troglodytes*, **B**. Three-population model: (*i*) *P. t. ellioti* (Rainforest), (*ii*) *P. t. ellioti* (Ecotone), (*iii*) *P. t. troglodytes*.

Aggregate ENMs were produced by averaging values from 100 replicate iterations of the data for both the two- and three-population models. These ENMs are shown in Figure 3 and are displayed using a logarithmic scale ranging from 0, corresponding to unsuitable habitat (cooler colors), to 1, corresponding to most suitable habitat (warmer colors). Figure 3A shows ENMs for the two-population model, which separates *P. t. ellioti* from *P. t. troglodytes* [27]. Figure 3B shows ENMs assuming a three-population model, which subdivides *P. t. ellioti* into two populations [27], one that inhabits the rainforests of western Cameroon and a second population that inhabits the ecotone of central Cameroon.

### Testing model performance

Table 1 shows the AUC (area under the curve) values for ENMs of each population under present conditions. For evaluating the robustness of an ENM, AUC values greater than 0.9 are considered to be ”very good” at describing a population’s niche, while AUC values of 0.7-0.9 are considered to be “good”, and less than 0.7 are classified as being “uninformative” [28]. All ENMs produced in this study performed better than ENMs produced by random associations between species presence and the environmental variables (AUC of 0.5). All empirical AUC values were greater than 0.94, suggesting that the ENM for each population was highly informative and described suitable habitats that corresponded very well with the environmental conditions found at presence localities recorded for each population considered in the study.

**Table 1:** **Average AUC values for each ecological niche model (average of 100 replicates)**

	**2-population model**		**3-population model**		
	***P. t. ellioti***	***P. t. troglodytes***	***P. t. ellioti*** **(Rainforest)**	***P. t. ellioti*** **(Ecotone)**	***P. t. troglodytes***
**AUC**	0.942	0.940	0.951	0.989	0.944
**Standard deviation**	0.036	0.037	0.045	0.011	0.041

Additional file 1 shows the results of the Maxent jackknife tests and the resulting percent contribution of each environmental predicting factor for both the two- and three-population models. In the two-population model, 44.5% of the ENM for *P. t. troglodytes* was defined by maximum temperature, and another 30% of the habitat was described by precipitation variables. The ENM for *P. t. troglodytes* in the three-population model showed similar trends in suitable habitat. In both the two- and three-population model, optimal habitat for *P. t. troglodytes* is relatively uniform moist rainforest. In contrast, *P. t. ellioti* occupies a much broader range of suitable habitat, including moist rainforest, woodlands, and open savanna, with more than 80% of the ENM for *P. t. ellioti* defined by trends in slope, temperature seasonality, tree cover, and precipitation.

Subdividing the *P. t. ellioti* population into groups located in northwest Cameroon and the ecotone revealed marked contrast in the habitats occupied by each population. Over 30% of the ENM for *P. t. ellioti* (Rainforest) is described by slope, and measures of precipitation and temperature seasonality described an additional 50% of their suitable habitat. The distribution of *P. t. ellioti* (Rainforest) is currently limited to the Cameroon Highlands and the Bakossi Mountains, where elevational gradients are prominent and the neighboring coastal region experiences high precipitation. The ENM for *P. t. ellioti* (Ecotone) describes an entirely different habitat, with high AUC values and the lowest standard deviation. Many variables contribute to describing from 5-12% of the suitable habitat of the ENMs for this population. Collectively these variables describe a suitable habitat for *P. t. ellioti* (Ecotone) that has greater variation in tree cover and that is drier, warmer, and more variable throughout the year compared to the optimal habitat of *P. t. ellioti* (Rainforest). Although humans may be expected to strongly influence wildlife distributions, human population density contributed little compared to landscape variables in defining the habitat of any chimpanzee population considered in this study.

Response curves for each of the environmental predicting factors were created for each ENM using Maxent. These graphs show the range of values for each factor that are most important for describing the suitable habitat of the population in question. Many environmental predicting factors contributed to differences in the optimal habitat of each population. For example, slope was found to be one of the most important factors that differentiate the habitat of *P. t. ellioti* (Rainforest) versus *P. t. ellioti* (Ecotone) from the habitat of *P. t. troglodytes* in southern Cameroon. Specifically, at slopes greater than 15 degrees the probability of suitable habitat was greater than 90% for *P. t. ellioti* as a whole as well as when the subspecies was subdivided into *P. t. ellioti* (Rainforest) and *P. t. ellioti* (Ecotone). The ENM of *P. t. ellioti* (Ecotone) also showed elevated gain in regions with sparse tree cover and less gain within areas of dense tree cover compared to *P. t. ellioti* (Rainforest)*.* Overall, individuals of *P. t. ellioti* (Rainforest) appear to occupy steep, densely forested areas. These habitats also experience high levels of precipitation throughout the year with a pronounced increase in precipitation from May – October. In contrast, *P. t. ellioti* (Ecotone) appears to occupy a wider breadth of habitats that include both forest and savanna, and they likely experience more seasonal variation in terms of temperature and precipitation throughout the year.

### Comparison of ENMs under present conditions

Table 2 shows values for the Schoener’s *D* test statistic [29] and the *I* test statistic [30] from the pairwise niche comparison tests for the two- and three-population models calculated using ENMtools [29]. The two-population model revealed that the niches occupied by *P. t. ellioti* and *P. t. troglodytes* are highly divergent from each other (p < 0.001). In addition, the three-population model that further subdivides *P. t. ellioti* into two subpopulations revealed that *P. t. ellioti* also occupies two significantly different niches that are located in the northwest of Cameroon and in the central Cameroon ecotone, respectively (p < 0.001). Both *P. t. ellioti* populations occupy niches that are significantly different from the niche occupied by *P. t. troglodytes* in southern Cameroon (p < 0.001). These observations align wellwith the inferred population genetic structure of chimpanzees currently occupying Cameroon and eastern Nigeria [27].

**Table 2:** **ENMtools Niche overlap test**

		**Schoener’s** ***D***				***I***			
**Model**	**Comparison**	**Observed**	**Null mean**	**Null SD**	***p***	**Observed**	**Null mean**	**Null SD**	***p***
2-population	Pte* and Ptt**	0.152	0.735	0.026	<0.001	0.405	0.937	0.011	<0.001
3-population	*Pte* (Ecotone) and *Ptt*	0.087	0.740	0.026	<0.001	0.257	0.935	0.013	< 0.001
	*Pte* (Rainforest) and *Ptt*	0.124	0.725	0.027	< 0.001	0.368	0.935	0.012	< 0.001
	*Pte* (Ecotone) and *Pte* (Rainforest)	0.113	0.759	0.024	< 0.001	0.341	0.943	0.010	< 0.001

*Pte (*Pan troglodytes ellioti*)

**Ptt (*Pan troglodytes troglodytes*)

### ENMs under climate change scenarios

**Figure 4:**
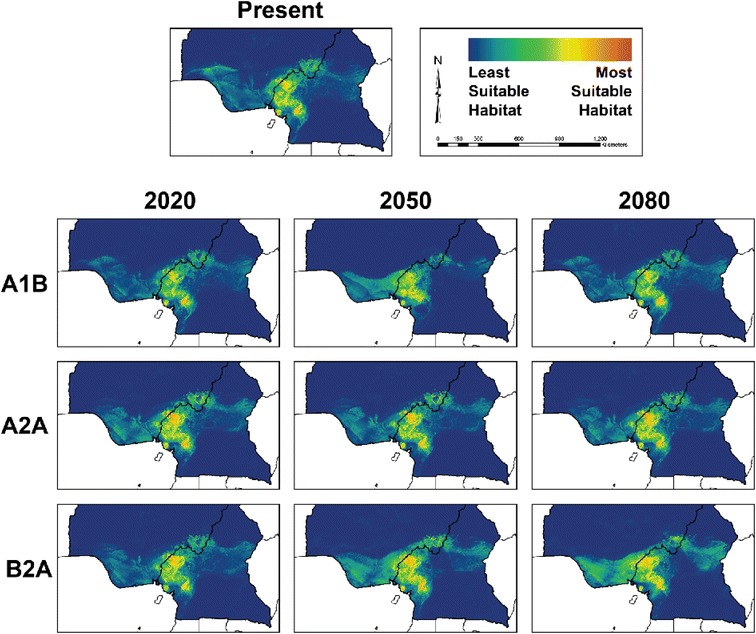
**Ecological niche models for** ***P. t. ellioti*** **(Rainforest) under scenarios of climate change.** Final ecological niche models produced by Maxent for *P. t. ellioti* (Rainforest) under each of the three climate scenarios tested. Warm colors show most suitable habitat, while cold colors show less suitable habitat.

**Figure 5:**
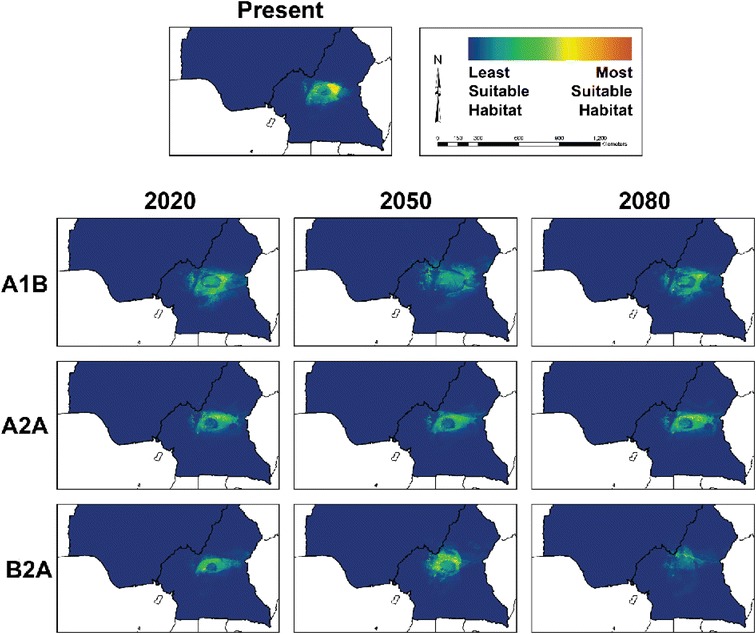
**Ecological niche models for** ***P. t. ellioti*** **(Ecotone) under scenarios of climate change.** Final ecological niche models produced by Maxent for *P. t. ellioti* (Ecotone) under each of the three climate scenarios tested. Warm colors show most suitable habitat, while cold colors show less suitable habitat.

Models of suitable habitat for chimpanzee populations under climate change scenarios were developed for the two populations of *P. t. ellioti. P. t. troglodytes* was excluded from these projections because the range of this subspecies extends far outside the study area, and the resulting predictions would likely be inaccurate because such models would not fully represent the environmental variation that this subspecies can occupy. Figures 4 and 5 show ENMs for *P. t. ellioti* subdivided into the *P. t. ellioti* (Rainforest) and *P. t. ellioti* (Ecotone) populations, respectively. Model performance for these ENMs under the various climate change scenarios was evaluated using AUC values (Additional file 2).

Figure 4 and Figure 5 show ENMs that are displayed on a logarithmic scale where 0 corresponds with unsuitable habitat (cooler colors) and 1 corresponds to suitable habitat (warmer colors). Figure 4 shows ENMs for *P. t. ellioti* (Rainforest) for years 2020, 2050, and 2080 under the A1B, A2A, and B2A climate scenarios [31], respectively. The A1B scenario describes an integrated world with balanced use of fossil fuels and non-fossil fuels, and human population growth followed by a gradual decline. The A2A scenario describes a heterogeneous or divided world where human population growth is continuous and countries are focused on preserving their local identities. The B2A scenario describes a heterogeneous or divided world where human population growth is continuous (but slower than A2A) and there is a local/regional focus on environmental protection [32]. Compared to the ENM for *P. t. ellioti* (Rainforest) under present conditions, the ENMs under these three climate scenarios suggests that *P. t. ellioti* (Ecotone) is unlikely to experience major shifts, contractions, or expansions of their suitable habitat through year 2080. Figure 5 shows the projected ENMs for *P. t. ellioti* (Ecotone) for years 2020, 2050, and 2080 under the A1B, A2A, and B2A climate scenarios [31], respectively. Overall, each of the tested climate scenarios used in this study suggest that optimal habitat for *P. t. ellioti* currently living in the ecotone habitat will be reduced by year 2020, and that the remaining optimal habitat for this population will become less suitable over time.

## Conclusions

Comparison of ENMs under present conditions suggests that *P. t. ellioti* and *P. t. troglodytes* occupy significantly different habitats (p < 0.001) (Figure 3 and Table 2). Overall, the optimal habitat for *P. t. troglodytes* in southern Cameroon is relatively uniform and mostly composed of moist rainforest. In contrast, the optimal habitat of *P. t. ellioti* is characterized by a higher degree of environmental variation and includes mountainous rainforest, lowland rainforest, woodlands, and savanna. Further subdividing the range of *P. t. ellioti* into two subpopulations improved ENM performance as evaluated by AUC values (Table 1). In addition, there appear to be two major niches occupied by *P. t. ellioti*: one subpopulation, *P. t. ellioti* (Rainforest), that occupies forested habitat in the mountains located in northwest Cameroon and a second subpopulation, *P. t. ellioti* (Ecotone), that occupies the forest-woodland-savanna ecotone in central Cameroon. These two areas were shown to be significantly different from each as well as from the optimal habitat occupied by *P. t. troglodytes*, as determined other by niche comparison tests (p < 0.001) (Table 2). Major differences in the two *P. t. ellioti* habitats include a steep altitudinal gradient and higher annual precipitation in the northwest and reduced forest cover with more distinct fluctuations in temperature and precipitation throughout the year in the ecotone.

The extent of the optimal habitats for each of these three populations correspond with the distribution of the three genetically distinct populations of chimpanzees that have been inferred to exist across the study area [27]. These ENMs suggest that (*i*) a relationship exists between environmental variation and the population genetic structure of chimpanzees across the study area, and (*ii*) that the Sanaga River is unlikely to be the only factor that contributes to the separation of *P. t. ellioti* from *P. t. troglodytes*. These results provide an ecological basis for the assertion that environmental variation across the region may be driving local adaptation. This is particularly compelling when coupled with the findings of two related studies that found that simple allopatric speciation is unlikely to explain the observed patterns of chimpanzee genetic diversity [27], and that a clear association exists between spatial patterns of genetic differentiation and habitat variation [33]. Taken together, these studies propose that populations of chimpanzees in Cameroon and Nigeria may be following a pattern of isolation-by-environment [34]. Furthermore, these findings suggest that environmental variation may also contribute to generating genetic variation within *P. t. ellioti*, as this subspecies occupies two fundamentally different niches in two different areas of Cameroon. The distribution of these two habitats corresponds very precisely with the inferred distribution of the two *P. t. ellioti* demes [27], which suggests that adaptation to different niches may play a role in the diversification of chimpanzee subspecies.

Recognizing that a positive relationship might exist between environmental and genetic variation in the distribution of chimpanzees also has important implications for broadening understanding about the puzzling distributions of other primates proposed also to be influenced by the Sanaga River, including *Mandrillus leucophaeus*/*M. sphinx*, *Cercopithecus erythrotis*/*C. cephus*, *C. nictitans martini*/*C. n. nictitans*, and *C. pogonias pogonias*/*C. p. grayi* [1,16,18,19,24]. These pairs of primates all occupy vastly different habitats and niches [1,18], which suggests that other factors along with, or instead of, the Sanaga River may be important in separating the distribution of these species, subspecies, and populations across the region. The role that environmental variation may have played in delimiting the distribution of these taxa remains uninvestigated. The existence of such a relationship would also be consistent with some insects [8], reptiles [9], and birds [10] in which the pronounced ecological gradient across Cameroon has been shown to be important in driving the population genetic structure of these species.

The African continent and central Africa in particular are predicted to be one of the most severely affected regions of the world by climate change [11-15]. Preliminary projections suggest that rainfall patterns will change dramatically in this region of Africa, which will result in significant alterations of forest and savanna habitats [35]. Models of global climate change also have been used to show that 30% of plant and animal species are at risk of extinction if the rise in mean global temperature exceeds 1.5°C - an increase that is nearly certain to occur under future climate scenarios [32,36]. Tropical forest taxa are widely believed to exist at a physiological optimum and their abilities to shift to new environmental conditions remains largely unknown [37]. Most of this evidence comes from amphibians, which suggests that even conservative projections of global warming will likely lead to widespread decline in amphibian populations across tropical regions [38,39]. Data regarding how climate change might affect mammals remain sparse, but climate induced range contractions have been linked to the loss of pockets of genetic distinctiveness in South African animals [40]. This suggests that it is reasonable to expect similar losses to occur in tropical forest mammals such as chimpanzees. Thus, the final step of this study involved examining how climate change might affect the optimal habitats of *P. t. ellioti* in the future. Specifically, the effects of climate change on the optimal habitats of *P. t. ellioti* were examined under the A1B, A2A, and B2A emission scenarios for years 2020, 2050, and 2080.

The predictions presented in this study suggest that the two distinct habitats occupied by *P. t. ellioti* will be affected differently. Little change is expected in mountainous, wet rainforests found in the northwest under any scenario across this time series. By contrast, optimal habitat in the ecotone is predicted to decline quickly under all scenarios by year 2020 and will disappear almost entirely under the worst-case scenario by 2080. These findings have important implications for understanding the conservation outlook for this subspecies. *P. t. ellioti* is the most endangered of all the chimpanzee subspecies, with only about 6,000 individuals estimated to persist across their entire range today and of which roughly half exist in this ecotone habitat [41]. Junker *et al.* [7] concluded that from the 1990s to the 2000s there had not been significant reductions in suitable environmental conditions for this subspecies, but the future predictions of our study indicate a drastic loss of suitable habitat by year 2020 followed by progressive degradation of suitable habitat for half of the range of *P. t. ellioti* due to climate change. However, future models presented in this study do not address the effects of continued human population growth, urban sprawl, agricultural development, and hunting, which are all expected to continue and accelerate across the region in coming years [41].

On a more positive note, there are several caveats to these sobering predictions. The models presented in this study do not take into account individual phenotypic plasticity or the potential for migration amongst these populations. In cases where loss of suitable habitat is likely to be significant, it is possible that these chimpanzees may be able to compensate and remain in their degrading habitat. They might also respond by migrating in order to track optimal environmental conditions, which is the simplest way that a population may respond to drastic changes in climate [42]. The effectiveness of migration to more optimal habitat relies on the availability of local suitable habitats for exploitation. Both micro- and macrorefugia have been shown to act as important reservoirs of genetic diversity in past large climatic events [43]. From a conservation point of view, migration corridors between protected areas are important for securing the long-term survival of taxa in regions where climate change is predicted to heavily modify the landscape [44]. The result of migration to new habitats is unknown, and should be studied in greater detail with the use of rigorous dispersal/demographic simulations [45]. One likely outcome is that pockets of genetic distinctiveness in *P. t. ellioti* will be lost along with their optimal ecotone habitat. To the extent that genetic distinctiveness is an important conservation goal, it is important that planning efforts take into consideration the effects of climate change on the distribution of optimal habitat, especially for *P. t. ellioti* (Ecotone).

It is currently unknown whether chimpanzees will exhibit niche conservatism or if they will adapt to changes in their habitats that result from climate change. Populations that exhibit strict niche conservatism over time may experience limited potential for range expansion and reduced dispersal opportunities since optimal habitat tracking may not be possible due to their divergent neighboring niches [46,47]. This possibility is especially plausible for *P. t. ellioti* (Ecotone) since this population exploits a niche that is completely divergent from the rainforest habitats of the neighboring *P. t. ellioti* (Rainforest) and *P. t. troglodytes* in southern Cameroon. This region of west central Africa is likely to experience drastic alterations that could lead to the loss of nearly all optimal chimpanzee habitat found in central Cameroon by 2080. Although the threats of hunting and habitat fragmentation by logging and agricultural plantations are immediate and are expected to have a large overall effect on chimpanzees in this region [41], the results of this study suggest that habitat loss due to climate change is a serious concern within our lifetimes and should not be ignored in conservation planning.

## Methods

This study was carried out in three phases. The first step involved generating ENMs for each of the two or three inferred chimpanzee populations, which required the acquisition and preparation of chimpanzee presence data from across the study region and the processing of environmental data to define niche dimensions. The second step involved: (*i*) using quantitative methods to determine whether optimal habitats for the inferred chimpanzee populations differed significantly from each other, and (*ii*) examining which variables made the largest contributions to differences between niches occupied by each population. The final step involved examining how climate change might affect the optimal habitat of each population in the future.

### Preparation of species occurrence data

Species occurrence data (Table 3 and Additional file 3) were obtained from www.ellioti.org [41] and from publications that involved sampling and/or observing wild chimpanzee populations across Cameroon and Nigeria from the late 1990s and early 2000s including both *P. t. ellioti* (N = 656) and *P. t. troglodytes* (N = 98) [48-51]. Occurrence data were compiled as geographic coordinates that indicated locations where chimpanzees were seen, heard, and/or indirect evidence of chimpanzee activity was found (nests, feeding sign, or tool use). Fecal and hair samples were shipped to the United States at ambient temperature, then stored at -20°C upon receipt. All samples were transported from Cameroon to the United States in full compliance with Convention of International Trade in Endangered Species of Wild Fauna and Flora (CITES) and Center for Disease Control (CDC) export and import regulations. Analysis of these samples was carried out with IACUC approval from the University at Albany – State University of New York.

**Table 3:** **Species occurrence data**

**Number of occurrences**	**Subspecies**	**Source**
634	*Pte* and *Ptt*	[41]
57	Pte* and Ptt**	[48]
19	*Ptt*	[49]
8	*Ptt*	[50]
7	*Ptt*	[51]

*Pte (*Pan troglodytes ellioti*).

**Ptt (*Pan troglodytes troglodytes*).

Duplicate occurrences with the same geographic coordinates were trimmed using ENMtools [29]. Second, an altitude map layer was created and used to trim duplicate occurrences that fell into the same grid cell of 1 km^2^. The remaining localities were projected in ArcMap 10 [52] for visual inspection to confirm that no more than one occurrence point fell into any one grid cell of the environmental data. Coordinates of occurrence data were then exported as a .csv formatted file for input into the Maxent software [53].

### Preparation of present environmental data

Environmental data used for this study are listed in Additional file 4. These environmental predicting factors were selected to best describe the habitat exploited by chimpanzees in Cameroon and Nigeria and included: (*i*) climatic factors and measures of climate stress such as isothermality and temperature seasonality [54,55], (*ii*) topographic factors such as elevation, slope, and percent tree cover [56,57], and (*iii*) anthropogenic presence as measured by human population density across the study area [58]. All environmental predicting factors were based on data gathered from 1994 to 2010, which corresponds to the time range of when all occurrence data were collected. Maps of environmental variables were transformed into the WGS 1984 coordinate projection because it preserves curvilinear features of the data and keeps it from being warped since the study area is within 15 degrees latitude of the equator [59]. This coordinate system also assured that the data retain compatibility with most publically available shapefiles for future projects and applications. All environmental layers used have a resolution of 30-arcseconds (about 1 km^2^), which was the finest resolution available at the time of publication for these layers at this multi-country scale.

### Maxent modeling under present conditions

ENMs were generated using a presence-only model implemented using the program Maxent [53]. This method was chosen for several reasons. Firstly, presence-only models, like Maxent, are useful because presence locality data are becoming more widely available for many taxa. Secondly, absence records are not widely available for chimpanzees and those that are available have often questionable accuracy due to the species’ large home ranges. Thirdly, a large comparative study has shown that the Maxent model outperforms other presence-only models such as GARP in many applications [60]. Finally, Maxent has also performed successfully in recent studies of other elusive and motile species [61-64].

The dataset of occurrence localities (described below) was divided into subsets for two- and three-populations from the inferred genetic structure shown by Mitchell *et al.* [27]. In the two-population model, occurrence data for *P. t. troglodytes* were separated from *P. t. ellioti* according to whether the point occurred north versus south of the Sanaga. The three-population model, included the group of presence points from *P. t. troglodytes* located south of the Sanaga, and the presence points from *P. t. ellioti* were subdivided into two groups. The first group was composed of presence points from *P. t. ellioti* west of the Mbam River, which is the main tributary of the Sanaga and demarcates the boundary of the ecotone. The second group of presence points was from *P. t. ellioti* located in the ecotone region found east of the Mbam River in central Cameroon.

Models were created using Maxent [53] with the default convergence threshold (10^-5^) and 100 cross-validated replicates. This cross-validation replicate process involved the random splitting of occurrence data into a number of equal-sized “folds” or groups where models were created leaving out one fold for each run. For each replicate, the excluded fold is used to evaluate the model [53].

### Testing model performance

Final models were evaluated using the area under the curve (AUC), which is a value widely used to measure model performance [60,65,66]. In brief, AUC values were created by comparing model performance to a random model of associations between presence localities and environmental predicting factors [66]. AUC values range from 0.5 to 1.0; with values close to 0.5 corresponding to a model that is no better at predicting an ecological niche than a random model, and a value of 1.0 corresponds to a model with a perfect fit. Values greater than 0.9 are ”very good”, 0.7-0.9 are “good”, and less than 0.7 are “uninformative” [28].

A jackknife test was also performed using Maxent to evaluate the individual contribution of each environmental predicting factor to each model. In the jackknife test, the contribution of each factor is tracked while the model is being created. Maxent does this by creating models with one predicting factor removed at a time and compares the jackknifed model gain to the gain of the complete model with all environmental predictors included. The factors that reduce the overall gain of the model when excluded become the most important [53].

### ENM comparison testing

Pairwise niche comparisons were carried out in ENMtools [29] to compare the degree of niche overlap between ENMs for both the two- and three-population models. For the three-population model, a round-robin comparison approach was implemented. For each comparison, two test statistics were calculated to estimate the degree of niche overlap: Schoener’s *D* [29] and the test statistic *I*, which was developed by Warren *et al.* [30]. Values of *D* and *I* are observed measurements of niche overlap that were used in the following analysis. In an ecological sense, Schoener’s *D* assumes that the suitability scores produced by Maxent are proportional to species abundance, whereas the test-statistic *I*, treats the two ENMs as probability distributions [29]. The significance of the observed *D* and *I* test statistics were evaluated in ENMtools by randomly partitioning a pooled set of occurrence data from two populations into two new datasets with the same number of occurrences as the original two populations. ENMtools then used these two new pseudo-populations to create ENMs using the Maxent algorithm. The *D* and *I* test statistics were then calculated to estimate the degree of overlap between the two new ENMs. A null distribution of values of *D* and *I* was created from 100 random pseudo-populations created using ENMtools. The observed values of *D* and *I* were then compared to the null distribution of *D* and *I* value*s* generated by random permutation. Significant deviations of observed values from the null values indicate that the niches occupied by the two populations under consideration are divergent [29]. The observed overlap values were compared to their respective null distributions using a student *t*-test in *R* [67].

### Climate change scenarios

The three different scenarios implemented in this study were A1B, A2A, and B2A (Additional file 5). The A1B scenario describes an integrated or homogenous world where economic growth is high and there is a balance between the use of fossil fuels and non-fossil fuels [32]. The A2A scenario describes a heterogeneous world with a steadily increasing human population throughout the century. The B2A scenario describes a divided world similar to the A2A scenario, but with each country or region working independently to reduce their emissions and the human population is steadily increasing throughout the century at a slower rate than the A2A scenario. These three scenarios describe a range of possible results of climate change over the next century that may play a role in the niche availability of chimpanzees in Cameroon and Nigeria.

### Preparation of data for future climate modeling

In order to model the distribution of these chimpanzee populations in the future, the following are required: 1) presence localities of chimpanzees in the present time, 2) a set of environmental variables used to describe their habitat for the present time, and 3) a matching set of environmental variables for each year under each climate scenario being explored. Since some measures of the environment cannot be predicted well using climate scenarios, due to other factors such as human disturbance, the projected models of distribution for the chimpanzee populations were created using only the climatic and topographic factors summarized in Additional file 4. For each scenario, bioclimatic files were created for each year being tested. In order to obtain the best mean values for each scenario, bioclimatic files were created for a number of global climate models (GCMs) and averaged for each scenario/year combination. The GCMs used for each scenario were obtained from www.ccafs-climate.org [31]. For any given scenario created by a GCM, minimum temperature (*tmin*), maximum temperature (*tmax*), and precipitation (*prec*) layers were obtained. Next, these three files were used to create the set of 19 bioclimatic files following the methods of Ramirez-Villegas and Bueno-Cabrera [68]. This was performed for each GCM for each climate scenario/year combination. Finally, environmental factors from each set of GCMs for a given scenario/year combination were averaged using ArcMap 10 for use in Maxent.

### Maxent modeling procedure under future climate scenarios

Modeling population distribution under climate change with Maxent is similar to modeling present distributions, and requires the same present occurrence coordinates and present environmental predictor variables [69-71]. However, modeling future climate scenarios additionally requires a matching sets of environmental variables for each time interval and climate scenario be specified for all populations under consideration. Maxent models the probability distribution for the present variables, as usual, to build a set of criteria that describes suitable habitat for the present time, and then examines future environmental variables for areas across the study area that best meet the species’ niche requirements. This analysis was completed by averaging 100 randomly-seeded replicates using the previously described cross-validation technique.

## Additional files

Additional file 1:
**Maxent Jackknife Test Results.** Results from Maxent jackknife tests for the average of 100 replicated runs for each population showing the percent contribution of each environmental variable to each ENM. Additional file 2:
**Testing Model Performance for Future ENMs.** Average AUC values for each ecological niche model (average of 100 replicates for each climate scenario. Additional file 3:
**Species Occurrence Map.** Map of occurrence data for *Pan troglodytes* in Cameroon and Nigeria. Additional file 4:
**Environmental Predicting Variables.** Table of environmental predicting variables used in ENMs. Additional file 5:
**Climate Scenario Aggregates.** Table showing organization of climatic variables for each included climate scenario.
