# Supplementary material for: Chimpanzee population structure in Cameroon and Nigeria is associated with habitat variation that may be lost under climate change
Source: BMC Evol Biol. 2015 Jan 21;15(1):2. doi: 10.1186/s12862-014-0275-z (PMC4314735; doi:10.1186/s12862-014-0275-z)
Supplement: Additional file 1: — Maxent Jackknife Test Results. Results from Maxent jackknife tests for the average of 100 replicated runs for each population showing the percent contribution of each environmental variable to each ENM. [file 12862_2014_275_MOESM1_ESM.docx]

|  | 2 Population  Model | | 3 Population  Model | | |
| --- | --- | --- | --- | --- | --- |
|  | *Pte* | *Ptt* | *Pte* (Rainforest) | *Pte* (Ecotone) | *Ptt* |
|  | **Percent Contribution** | | | | |
| Slope | **11.9** | 1.9 | **30.5**^†^ | 0.1 | 1.7 |
| Altitude | 3.3 | 2.1 | 1.0 | **10.4** | **3.2** |
| Annual Mean Temperature (Bio1) | 0.1 | 0.6 | 1.1 | 0.0 | 1.2 |
| Mean Diurnal Range (Bio2) | 0.9 | 2.5 | 1.3 | **7.4** | **2.8** |
| Isothermality (Bio3) | 1.1 | 1.4 | 1.4 | **6.4** | 1.8 |
| Temperature Seasonality (Bio4) | **24.0** | 3.1 | **10.9** | 4.5 | 1.9 |
| Max. Temp. of the Warmest Month (Bio5) | 0.7 | **44.5** | 1.1 | 0.9 | **44.5** |
| Min. Temp. of the Warmest Month (Bio 6) | 0.2 | 0.7 | 0.2 | 2.3 | 0.3 |
| Temperature Annual Range (Bio 7) | 5.5 | **3.9** | 1.0 | **7.0** | 2.6 |
| Mean Temp. of the Wettest Quarter (Bio8) | 0.1 | 0.4 | 0.9 | 0.0 | 0.1 |
| Mean Temp. of the Driest Quarter (Bio9) | 0.3 | 0.2 | 0.6 | 0.3 | 0.0 |
| Mean Temp. of the Warmest Quarter (Bio10) | 0.6 | 0.5 | 0.1 | **10.8** | 1.2 |
| Mean Temp. of the Coldest Quarter (Bio11) | 0.1 | 0.3 | 0.2 | 0.0 | 0.1 |
| Annual Precipitation (Bio12) | 0.2 | 0.8 | **13.4** | 0.2 | 1.9 |
| Precipitation of the Wettest Month(Bio13) | 2.3 | 0.1 | 1.4 | 1.6 | 0.1 |
| Precipitation of the Driest Month (Bio14) | 0.2 | **8.2** | 0.7 | 2.5 | **13.1** |
| Precipitation Seasonality (Bio15) | 0.9 | **17.9** | 0.4 | 0.0 | **12.4** |
| Precipitation of the Wettest Quarter (Bio16) | **9.7** | 0.4 | **14.8** | **8.9** | 1.0 |
| Precipitation of the Driest Quarter (Bio17) | 0.8 | **5.3** | 0.4 | **11.1** | **5.5** |
| Precipitation of the Warmest Quarter (Bio18) | 0.8 | 1.1 | 0.3 | **10.3** | 1.1 |
| Precipitation of the Coldest Quarter (Bio19) | **17.1** | **3.3** | **15.7** | 3.6 | 2.2 |
| Human Pop | 1.2 | 0.2 | 0.5 | 0.8 | 0.2 |
| Tree Cover | **18.3** | 0.7 | 2.1 | **10.7** | 1.1 |

†Bold values represent about the top 80% of contributing variables for each population.
