# Supplementary material for: Chimpanzee population structure in Cameroon and Nigeria is associated with habitat variation that may be lost under climate change
Source: BMC Evol Biol. 2015 Jan 21;15(1):2. doi: 10.1186/s12862-014-0275-z (PMC4314735; doi:10.1186/s12862-014-0275-z)
Supplement: Additional file 2: — Testing Model Performance for Future ENMs. Average AUC values for each ecological niche model (average of 100 replicates for each climate scenario. [file 12862_2014_275_MOESM2_ESM.docx]

| **Variable Type** | **Variable Name** | **Source** |
| --- | --- | --- |
| **Climatic**  **Factors** | Bio1 – Annual Mean Temperature | WorldClim;  [55] |
|  | Bio2 – Mean Diurnal Range |  |
|  | Bio3 – Isothermality |  |
|  | Bio4 – Temperature Seasonality |  |
|  | Bio5 – Max. Temp. of the Warmest Month |  |
|  | Bio 6 – Min. Temp. of the Warmest Month |  |
|  | Bio 7 – Temperature Annual Range |  |
|  | Bio8 – Mean Temp. of the Wettest Quarter |  |
|  | Bio9 – Mean Temp. of the Driest Quarter |  |
|  | Bio10 – Mean Temp. of the Warmest Quarter |  |
|  | Bio11 – Mean Temp. of the Coldest Quarter |  |
|  | Bio12 – Annual Precipitation |  |
|  | Bio13 – Precipitation of the Wettest Month |  |
|  | Bio 14 – Precipitation of the Driest Month |  |
|  | Bio15 – Precipitation Seasonality |  |
|  | Bio 16 – Precipitation of the Wettest Quarter |  |
|  | Bio 17 – Precipitation of the Driest Quarter |  |
|  | Bio18 – Precipitation of the Warmest Quarter |  |
|  | Bio19 – Precipitation of the Coldest Quarter |  |
| **Topographic**  **Factors** | Elevation | NASA SRTM;  [56] |
|  | Slope | Derived from above  In ArcMap 10  [52] |
|  | Percent Tree Cover | MODIS;  [57] |
| **Anthropogenic**  **Factor** | Human Population Density | ORNL Landscan;  [58] |
