# Supplementary material for: Chimpanzee population structure in Cameroon and Nigeria is associated with habitat variation that may be lost under climate change
Source: BMC Evol Biol. 2015 Jan 21;15(1):2. doi: 10.1186/s12862-014-0275-z (PMC4314735; doi:10.1186/s12862-014-0275-z)
Supplement: Additional file 3: — Species Occurrence Map. Map of occurrence data for Pan troglodytes in Cameroon and Nigeria. [file 12862_2014_275_MOESM3_ESM.png]

|  | ***P. t. ellioti* (Rainforest)** | | | | | |
| --- | --- | --- | --- | --- | --- | --- |
|  | **A1B** | | **A2A** | | **B2A** | |
| **Year** | **AUC** | **StdDev** | **AUC** | **StdDev** | **AUC** | **StdDev** |
| **2020** | 0.955 | 0.041 | 0.955 | 0.038 | 0.954 | 0.042 |
| **2050** | 0.955 | 0.038 | 0.957 | 0.036 | 0.956 | 0.034 |
| **2080** | 0.956 | 0.037 | 0.957 | 0.038 | 0.956 | 0.038 |
|  | ***P. t. ellioti* (Ecotone)** | | | | | |
|  | **A1B** | | **A2A** | | **B2A** | |
| **Year** | **AUC** | **StdDev** | **AUC** | **StdDev** | **AUC** | **StdDev** |
| **2020** | 0.988 | 0.011 | 0.989 | 0.013 | 0.988 | 0.013 |
| **2050** | 0.989 | 0.012 | 0.988 | 0.012 | 0.988 | 0.012 |
| **2080** | 0.988 | 0.011 | 0.988 | 0.010 | 0.987 | 0.013 |
