# Supplementary figures and images for: Chimpanzee population structure in Cameroon and Nigeria is associated with habitat variation that may be lost under climate change
Source: BMC Evol Biol. 2015 Jan 21;15(1):2. doi: 10.1186/s12862-014-0275-z (PMC4314735; doi:10.1186/s12862-014-0275-z)

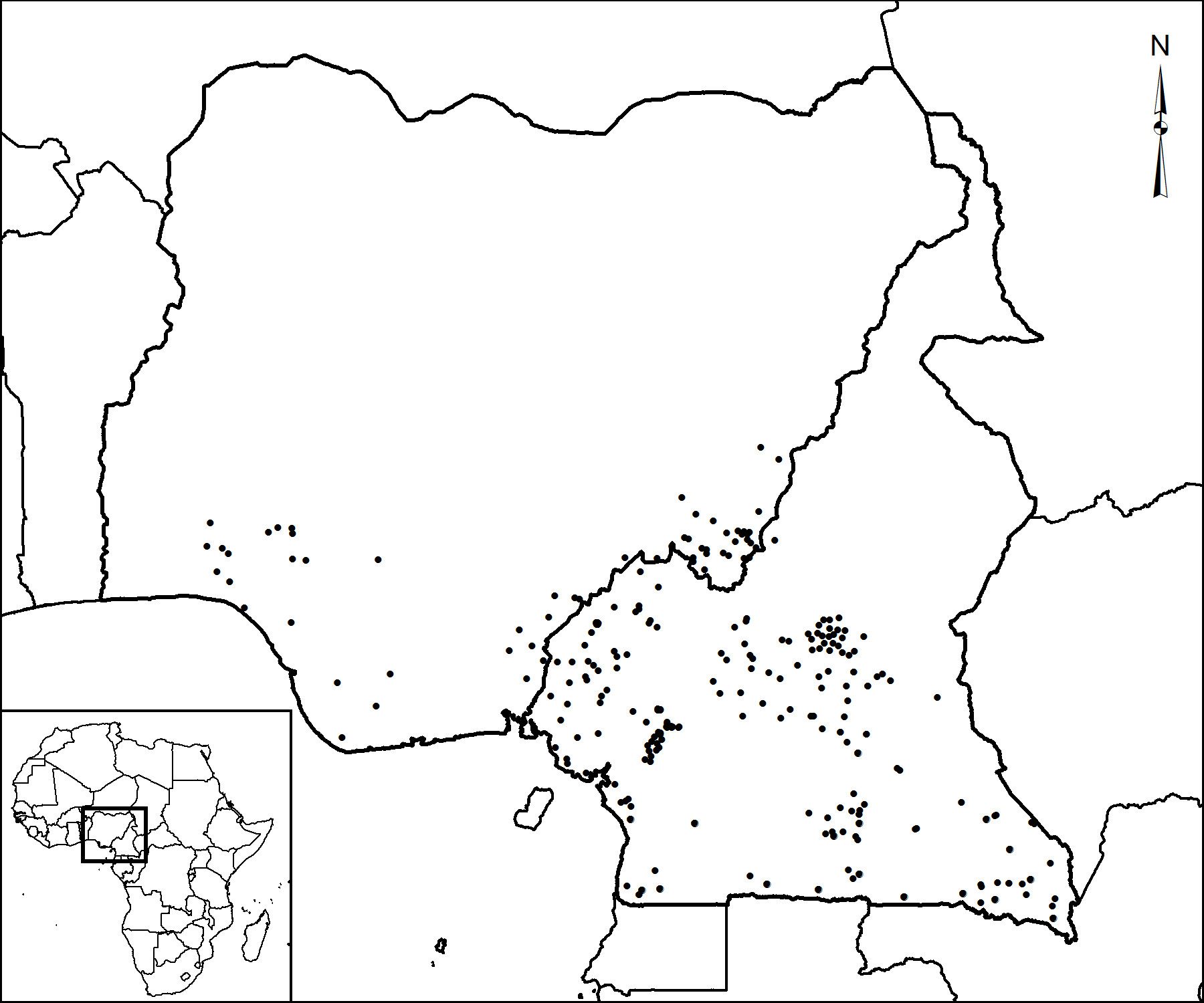

Supplement: Additional file 4: — Environmental Predicting Variables. Table of environmental predicting variables used in ENMs. [file 12862_2014_275_MOESM4_ESM.docx]
