# Supplementary material for: Chimpanzee population structure in Cameroon and Nigeria is associated with habitat variation that may be lost under climate change
Source: BMC Evol Biol. 2015 Jan 21;15(1):2. doi: 10.1186/s12862-014-0275-z (PMC4314735; doi:10.1186/s12862-014-0275-z)
Supplement: Additional file 5: — Climate Scenario Aggregates. Table showing organization of climatic variables for each included climate scenario. [file 12862_2014_275_MOESM5_ESM.docx]

| **Climate Scenario** | **Global Climate Models Included in Aggregate** | **Environmental Factors** | **Years** |
| --- | --- | --- | --- |
| **A1B** | CCCMA | Bioclimatics 1 - 19 | 2020, 2050,  and 2080 |
|  | CSIRO MK3 |  |  |
|  | IPSL CM4 |  |  |
|  | MPI ENCHAM5 |  |  |
|  | NCAR CCSM3 |  |  |
|  | UKMO HADCM3 |  |  |
| **A2A** | CCCMA |  |  |
|  | CSIRO MK3 |  |  |
|  | HCCPR HADCM3 |  |  |
|  | NIES99 |  |  |
| **B2A** | CCCMA |  |  |
|  | CSIRO MK3 |  |  |
|  | HCCPR HADCM3 |  |  |
|  | NIES99 |  |  |
